# Identification of phosphorylation proteins in response to water deficit during wheat flag leaf and grain development

**DOI:** 10.1186/s40529-018-0245-7

**Published:** 2018-12-08

**Authors:** Fei Luo, Xiong Deng, Yue Liu, Yueming Yan

**Affiliations:** 1 College of Life Science, Capital Normal University, Beijing, 100048 China; 2 Hubei Collaborative Innovation Center for Grain Industry (HCICGI), Yangtze University, Jingzhou, 434025 China

**Keywords:** Bread wheat, Phosphorylated proteins, Water deficit, Flag leaves, Developing grains

## Abstract

**Background:**

Wheat (*Triticum aestivum* L.) serves as important grain crop widely cultivated in the world, which is often suffered by drought stress in natural conditions. As one of the most important post translation modifications, protein phosphorylation widely participates in plant abiotic stress regulation. In this study, we performed the first comparative analysis of phosphorylated protein characterization in flag leaves and developing grains of elite Chinese bread wheat cultivar Zhongmai 175 under water deficit by combining with proteomic approach and Pro-Q Diamond gel staining.

**Results:**

Field experiment showed that water deficit caused significant reduction of plant height, tiller number, ear length and grain yield. 2-DE and Pro-Q Diamond gel staining analysis showed that 58 proteins were phosphorylated among 112 differentially accumulated proteins in response to water deficit, including 20 in the flag leaves and 38 in the developing grains. The phosphorylated proteins from flag leaves mainly involved in photosynthesis, carbohydrate and energy metabolism, while those from developing grains were closely related with detoxification and defense, protein, carbohydrate and energy metabolism. Particularly, water deficit resulted in significant downregulation of phosphorylated modification level in the flag leaves, which could affect photosynthesis and grain yield. However, some important phosphorylated proteins involved in stress defense, energy metabolism and starch biosynthesis were upregulated under water deficit, which could benefit drought tolerance, accelerate grain filling and shorten grain developing time.

**Conclusions:**

The modification level of those identified proteins from flag leaves and grains had great changes when wheat was suffered from water deficit, indicating that phosphoproteins played a key role in response to drought stress. Our results provide new insights into the molecular mechanisms how phosphoproteins respond to drought stress and thus reduce production.

**Electronic supplementary material:**

The online version of this article (10.1186/s40529-018-0245-7) contains supplementary material, which is available to authorized users.

## Background

As one of the most important grain crops, wheat (*Triticum aestivum* L.) is widely cultivated in the world due to its value as a stable source of saccharides and proteins, and its ability to adapt to different surviving environment. However, most agricultural areas where wheat mainly spread over strongly depend on natural rainfall, including almost all arid and semiarid areas (Cai et al. 2012). In recent years, climate changing, especially global warming, has exacerbated the effects of drought stress on crop production. In general, temperature rising 1 °C can produce a decrease in yield of up to 10% (Lobell et al. 2011).

Drought is one of the most common abiotic stresses, which significantly affects crop production (Fedoroff et al. 2010). Drought stress induces a series of physiological and biochemical changes in plants, strongly interferes cellular homeostasis and causes morphological changes, e.g. repression of cell growth and photosynthesis, and activation of respiration. Moreover, when two experimental groups are respectively exposed to water sufficiency and water deficiency conditions during grain filling, the results of stored carbon remobilization in wheat are apparently different: one group is senescence and the other accelerates grain filling (Yang et al. 2000, 2001). However, telluric plants have developed specific mechanisms to response and tolerance to short- and long-term adverse environments, particularly to drought (Harb et al. 2010). For example, plants can accumulate reactive oxygen species (ROS) and proteins to enhance drought tolerance (Zhu 2002). In addition, when encountered drought stress, plant root caps synthesize abscisic acid (ABA) to trigger a signaling cascade in guard cells, and then leads to stomatal closure and reduction of water loss (MacRobbie 1998).

Plant leaves are the largest organ of photosynthesis with highest photosynthetic efficiency, providing an important source of carbohydrate for developing grains. Therefore they serve as the ultimate yield-limiting factor (Slafer et al. 1990). As reported, the contribution of wheat flag leaves to grain yield is up to 41–43% (Araus and Tapia 1987). However, photosynthesis is particularly sensitive to water deficit and the photosynthetic rate is negatively related to water content (Lawlor and Cornic 2002). Meanwhile, drought causes foliar stoma limitation and reduces air exchanges (Cornic 2000). In addition, metabolic repair can be suppressed by drought, causing the limitation of photosynthesis and significant reduction of carbon assimilation and utilization capacity (Reddy et al. 2004). Grain endosperm in wheat consists of about 70% starch and 14% proteins, which are the major determinant of yield and quality (Johansson et al. 2001; Donner and Mesdag 2000). These reserve substances are rapidly synthesized and accumulated after flowering, and this process involves lots of genes and enzymes (Cao et al. 2015; Yu et al. 2016). At least four types of enzymes, phosphorylated by other kinases in the amyloplast of higher plant endosperm, are involved in starch biosynthesis such as branching enzymes, ADP glucose pyrophosphorylase (AGPase), starch synthases (SS) and debranching enzymes (DBE).

Protein phosphorylation, as one of the most important protein posttranslational modifications (PTMs), is often reversible and transient (Hunter and Karin 1992). Serine, threonine, and tyrosine (Ser/Thr/Tyr) are the key phosphorylated modification sites in proteins (Stock et al. 1989; Cohen 2002; Aivaliotis et al. 2009), Plenty of enzymes perform their functions in signaling pathways, protein abundance or activity regulation via protein phosphorylation (Engelsberger and Schulze 2012). The phosphorylation of some key proteins involved in ABA signal transduction pathway contributes to cellular growth and abiotic stress response (Thingholm et al. 2009; Zhang et al. 2014a). Protein phosphorylation also regulates many essential biochemical processes such as DNA transcription, protein translation, and energy metabolism (Kersten et al. 2009).

In recent years, extensive phosphoproteomic analyses in different plant species were performed to explore the molecular basis of plant growth and abiotic stress responses such as *Arobidopsis thaliana* (Umezawa et al. 2013), *Brachypodium distachyon* (Lv et al. 2014a, b; Yuan et al. 2016), rice (Chang et al. 2012), wheat (Zhang et al. 2014a, b; Dong et al. 2015; Lv et al. 2016), maize (Hu et al. 2015a, b) and barley (Horie et al. 2011). Particularly, Pro-Q Diamond phosphoprotein staining can specially bind to the part of phosphate of phosphoproteins as well as phosphoamino acids. Therefore it serves as an efficient mean to rapidly detect phosphorylated proteins (Silva-Sanchez et al. 2015). Through Pro-Q Diamond staining, protein phosphorylated modification was widely present in germinating seeds (Dong et al. 2015), developing grains (Guo et al. 2012; Zhang et al. 2014b), starch granules (Chen et al. 2014, 2017; Cao et al. 2015) and seedling leaves response to salt stress (Lv et al. 2016). Various abiotic stresses such as drought and salt can induce significant changes in protein phosphorylated levels (Zhang et al. 2014a; Bian et al. 2017). Thus, protein phosphorylation plays key roles in wheat growth and development, and starch biosynthesis as well as in response to various adverse environments. However, to our knowledge, the phosphorylated protein characterization in wheat developing flag leaves and grains and their potential roles in regulating grain development and yield formation are still not clear.

In this study, we aim to characterize the phosphorylated proteins in wheat developing flag leaves and grains at 20 days postanthesis (DPA) under field water-deficit treatment and to reveal the potential roles of phosphorylated proteins involved in drought stress response and grain development. Our results provide new evidence for further understanding the molecular mechanisms of flag leaves and grains synergistically respond to drought stress.

## Materials and methods

### Wheat materials, field drought treatments and sampling

Chinese elite bread wheat cultivar Zhongmai 175 (*Triticum aestivum* L.) was used as material and planted in the experimental station of China Agricultural University (CAU), Wuqiao, Hebei Province (116°37′23″E and 37°16′02″N) during 2016–2017 wheat growing season. Basic fertility of the experiment plot was measured before sowing. The organic matter, total nitrogen, hydrolysable nitrogen, available phosphorus and available potassium in the topsoil (0–20 cm) of the experimental plots were 12.1 g kg^−1^, 1.0 g kg^−1^, 106.7 mg kg^−1^, 33.8 mg kg^−1^ and 183.4 mg kg^−1^, respectively. Total precipitation was 128 mm at 2016–2017 wheat growing season, lower than the annual mean amount (130–180 mm).

Field experiments contained two irrigation treatment groups in three biological replicates: drought treatment group without irrigation after sowing and control group with two times of irrigation after sowing watered 75 mm at the jointing and anthesis stage, respectively. Each experimental plot was 8 × 4 m with rows spaced at 0.16 m and 1 m interval between control and treatment groups was maintained to minimize the effects of adjacent plots. The watering amount was measured by a flow meter. Soil samples at 20 DPA were collected from 0 m increments to a depth of 2 m with a soil corer and soil water content was determined by using the oven-drying method (Gardner 1986).

Before sowing, relative water content of target field was irrigated to 80.5% of the field water capacity at 0–200 cm soil layer, and then seeds were sowed when soil water content got to 80% (Chu et al. 2016). The Zadoks scale was used to categorize crop developmental stages (Zadoks et al. 1974). The plants were marked after flowering, and then flag leaves and developing grains at 20 DPA in three biological replicates were harvested, and immediately immerged into liquid nitrogen prior to use. After maturity, main agronomic traits and grain yield were tested.

### Protein extraction

Proteins from flag leaves and grains were extracted according to the previous report (Zhang et al. 2014b) with minor modifications. Leaves and grain samples (each 0.5 g) from three biological replicates were ground into fine powder by liquid nitrogen, then mixed with 1 mL of extraction buffer consisting of 50 mM Tris–HCl (pH 8.0), 0.1 M KCl, 5 mM EDTA and 30% sucrose. PhosSTOP phosphatase inhibitor cocktail (1 tablet/10 mL, Roche, Basel, Switzerland) was added. After placing for 15 min, the samples were shaken vigorously for 30 min at room temperature, and then centrifuged to divide mixture. Protein supernatants were precipitated with a one-quarter volume of cold 10% trichloroacetic acid at − 20 °C for 4 h. The precipitated proteins were rinsed with cold acetone (− 80 °C) and then centrifuged three times at 13,000*g* for 10 min. After frozen dryly, 300 µL solubilization buffer was added and placed at room-temperature for 4 h. The protein concentrations were quantitated by 2-D Quant Kit (Amersham Bioscience, USA) and the protein solution was stored at − 80 °C for later use.

### 2-DE

The differentially accumulated proteins (DAPs) from three biological replicates were separated by two-dimensional electrophoresis (2-DE) based on Lv et al. (2014b). The isoelectric focusing (IEF) was performed using 18 cm linear gradient IPG strip (GE Healthcare, Little Chalfont, UK). After IEF, the equilibration solution (1% DTT) was applied to equilibrate the strips for 15 min and then the second equilibration was performed with 2.5% w/v iodoacetamide. Subsequently, the strips were loaded on the top of 12% SDS–polyacrylamide gels for SDS–PAGE, and 2-DE gels were stained by Coomassie blue. The ImageMaster 2D Platinum 7.0 (GE Healthcare, USA) was used to analyze the images and only those with significant and biological reproducible changes (abundance variation at least twofold, Student’s *t* Test, *p* < 0.05) were used as DAP spots.

### Detection of phosphorylated proteins by Pro-Q Diamond gel staining

2-DE gels were stained with Pro-Q Diamond (Invitrogen, USA) to detect the different level of phosphoproteins by the previous method (Zhang et al. 2014a). The gels were fixed twice for 30 min/each time and washed three times with ddH_2_O for 10 min/each time. Subsequently, the gels were incubated in Pro-Q Diamond staining in darkness for 2 h and destained with 20% acetonitrile in 50 mM sodium acetate (pH 4.0) four times (30 min each). The gels were scanned on a TyphoonTM 9400 scanner (GE Healthcare, USA) with a 532 nm excitation laser and a 610 nm long pass filter. The gels were stained with CBB to visualize total phosphoproteins after fluorescent image acquisition. The phosphorylated proteins were identified through comparison between 2-DE gels and Pro-Q Diamond gel staining results.

### MALDI-TOF/TOF-MS

The DAP and phosphorylated protein spots identified were manually excised from the 2-DE gels and digested with trypsin as the reported method (Lv et al. 2016). Matrix-assisted laser desorption/ionization time-of-flight/time-of-flight mass spectrometer (MALDI-TOF/TOF-MS) was used to identify the DAPs by ABI 4800 Proteomics Analyzer. The MS/MS spectra were searched in Viridiplantae (green plant) sequences in the nonredundant National Center for Biotechnology Information (NCBI) database and Triticum NCBI database. MASCOT software (ver. 2.1; Matrix Science, London, UK) was used and all searches were evaluated based on the significant scores obtained from MASCOT. A significance threshold of *p* < 0.05 was used, and the protein score CI% and total ion score CI% were both set to > 95%.

### Validation of phosphorylated proteins by Western blotting

Western blotting was performed to further verify the phosphorylated proteins identified by Pro-Q Diamond staining by using Anti-Phosphoserine/threonine/tyrosine monoclonal antibody from Abcam (Cat. No. SPM101, MA, USA) according to the previous report (Chen et al. 2014).

### Bioinformatics analysis

Protein function was classified based on the annotation from UniProt (Wang et al. 2015). The subcellular localization was predicted according to FUEL-mLoc Server (http://bioinfo.eie.polyu.edu.hk/FUEL-mLoc/), WoLF PSORT (http://www.genscript.com/wolf-psort.html), CELLO version 2.5 (http://cello.life.nctu.edu.tw/), Plant-mPLoc (http://www.csbio.sjtu.edu.cn/bioinf/plant-multi/) and UniProtKB. Biological statistic analyses were performed by SPSS statistics software (ver. 19.0; SPSS Inc., Chicago, IL, USA) to intuitively view the results. The relative expression quantity of proteins between well-watered and water-deficit group was represented as the average of three replicates and compared by one-way ANOVA. Cluster 3.0 and visualized with Java TreeView were used for protein clustering analysis. Hierarchical clustering of the expression profiles was carried out based on the log transformed fold change values of protein spots and average linkage clustering method with Euclidean distance similarity metric was used (Wang et al. 2017). Meanwhile, NetPhos 3.1 Server (Blom et al. 1999) (http://www.cbs.dtu.dk/services/NetPhos/) was used to predict phosphorylation sites among the identified phosphorylated proteins.

## Results

### Soil water content, agronomic trait and yield changes under water deficit

**Fig. 1:**
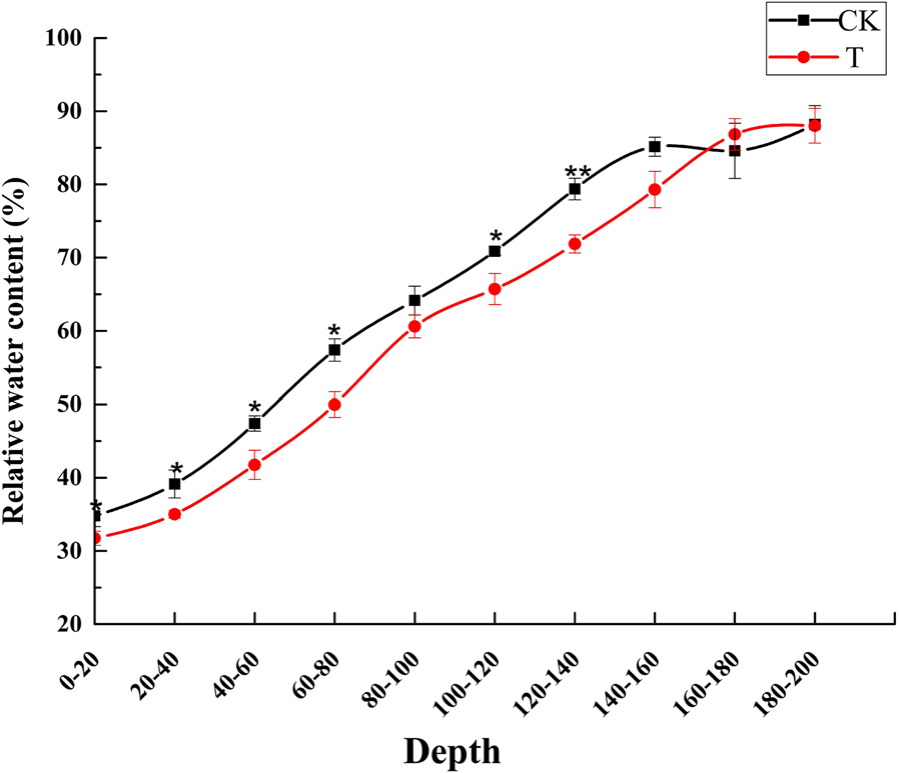
Soil relative water content at 20 DPA. Soil relative water content was measured at 2 m depth of soil in the control (CK) and drought treatment (T) groups. Statistically significant differences compared to the control were calculated based on an independent Student’s t-tests. Asterisks indicate significant different (*p < 0.05; **p < 0.01). Error bars indicate standard errors of three biological replicates

The changes of relative soil water content between 0 and 2 m depths in the control group and drought treatment groups at 20 DPA were shown in Fig. 1. The results showed that the relative soil water content of 0 to 1.4 m depth had obvious discrepancy between the control and drought treatment. Thus, severe drought in the treatment group occurred in the 0–60 cm soil layer, and mild drought in the 60–140 cm soil layer based on the grade of agricultural drought in GB/T 32136-2015 (Lv et al. 2015).

**Fig. 2:**
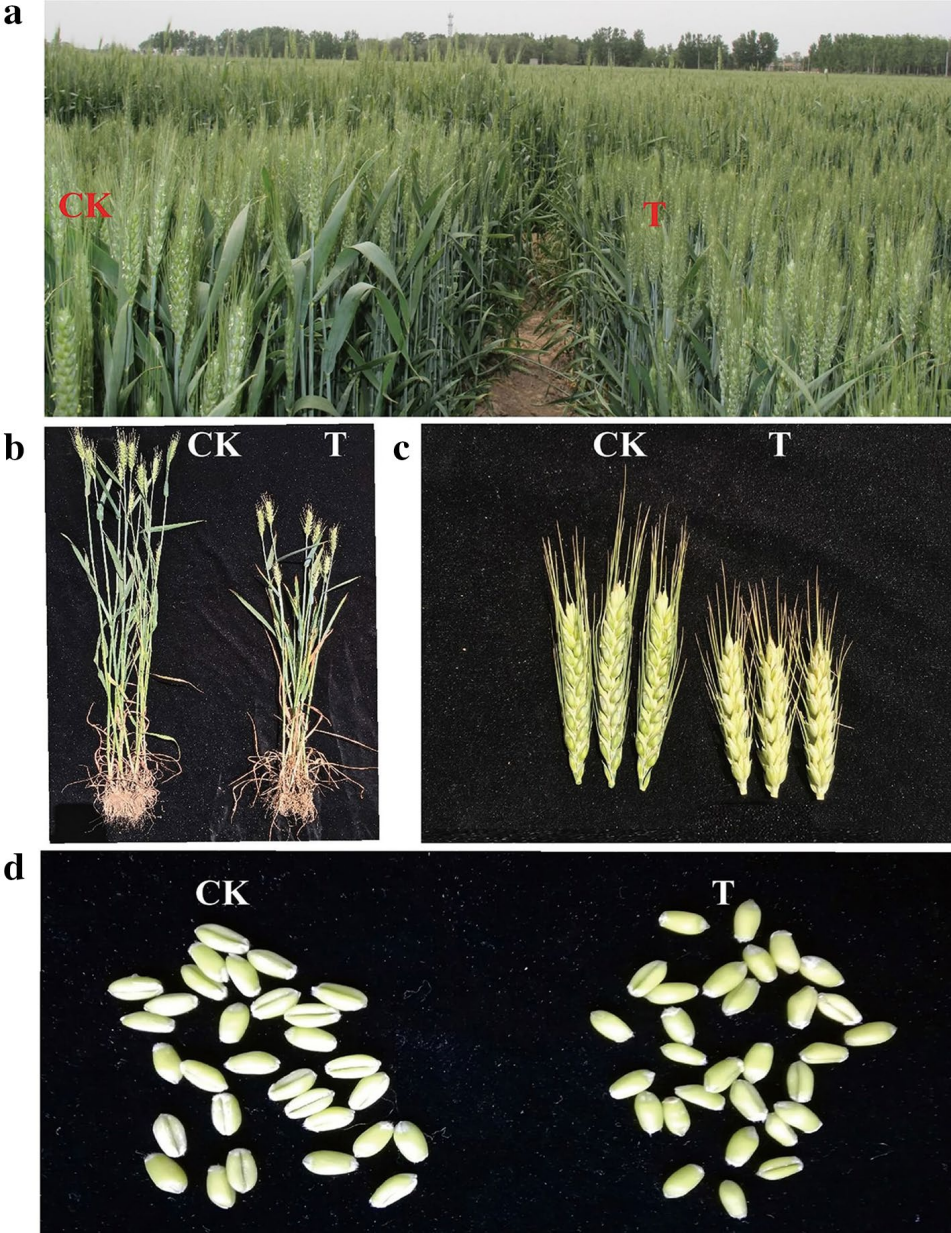
Plant phenotypes under stress treatment and CK. **a**, **b** Plant height compared at 20 DPA between CK and drought treatment groups. **c** Ear length at 20 DPA under drought treatment. **d** Grain appearance at 20 DPA under drought treatment

Water deficit resulted in significant changes of main agronomic traits and grain yield. In the field, plant leaves became yellower and plant height was more lethargic (Fig. 2a, b) and wheat ears and grains were smaller and yellower in the treatment group than the control group (Fig. 2c, d). Thus, plant height, ear length, tiller numbers, grain number per spike and 1000-grain weight were significantly decreased. Ultimately, drought treatment caused significant reduction of grain yield, up to 19.23% (Additional file 1: Table S1).

### Identification of differentially accumulated proteins (DAPs) responsive to water deficit in flag leaves and developing grains

**Fig. 3:**
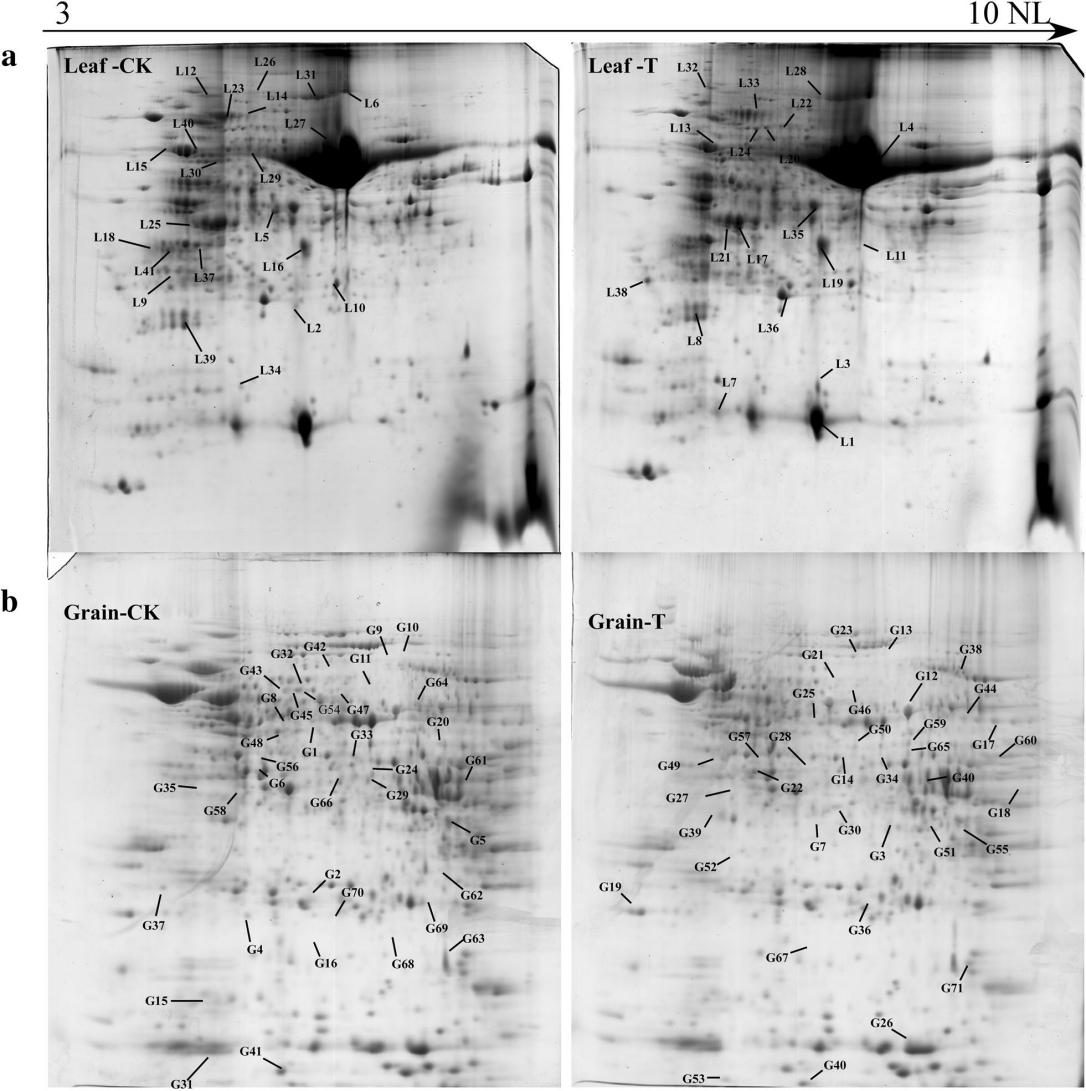
2-DE gel images of flag leaves and developing grains at 20 DPA in Zhongmai 175 under drought stress. **a** Flag leaf 2-DE gels of CK and drought treatment. **b** Grain 2-DE gels of CK and drought treatment

**Fig. 4:**
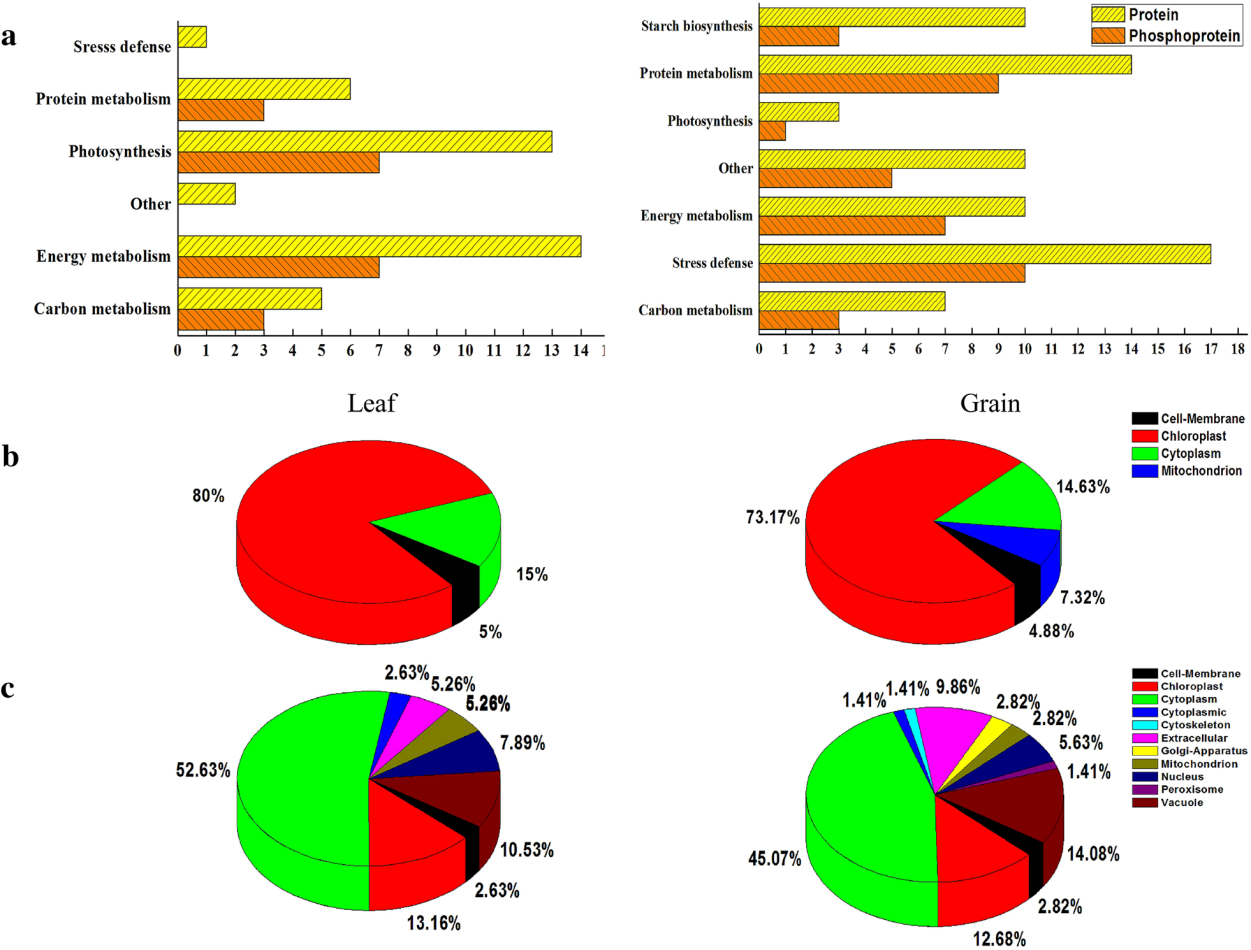
Functional classification, subcellular localization and Venn diagram analysis of DAPs from flag leaves and developing grains in Zhongmai 175. **a** Functional classification of proteins in flag leaf and developing grain. **b** Subcellular localization of all proteins (right) and phosphoproteins (left) that we had identified in leaves. **c** Subcellular localization of all proteins (right) and phosphoproteins (left) that we had identified in developing grains

2-DE and tandem mass spectrometry identified 41 and 71 DAP spots in response to water deficit, which respectively contained 36 and 64 unique proteins at 20 DPA in flag leaves and grains (Fig. 3). The detailed information and peptide sequences of the identified proteins are listed in Additional file 1: Tables S2A, B and S3A, B. The function annotation from UniProt showed that 100 unique proteins were classified into 7 functional categories: carbon metabolism, stress defense, energy metabolism, photosynthesis, protein metabolism, storage substance biosynthesis. In the flag leaves, the DAPs were mainly involved in energy metabolism (34.17%), photosynthesis (31.70%) and protein metabolism (14.63%) while those in the developing grains were mainly related to stress defense (23.94%), protein metabolism (16.90%) and storage substance biosynthesis (16.90%) (Fig. 4a). Subcellular localization showed that most of the DAPs in flag leaves and grains were located in chloroplast (73.17%) and cytoplasm (45.07%), respectively (Fig. 4b, c). These results indicate that the foremost function of flag leaves is photosynthesis while grain proteins play important roles in abiotic stress defense and reserve substance synthesis.

### Characterization of phosphorylated proteins in the developing flag leaves and grains under water deficit

**Fig. 5:**
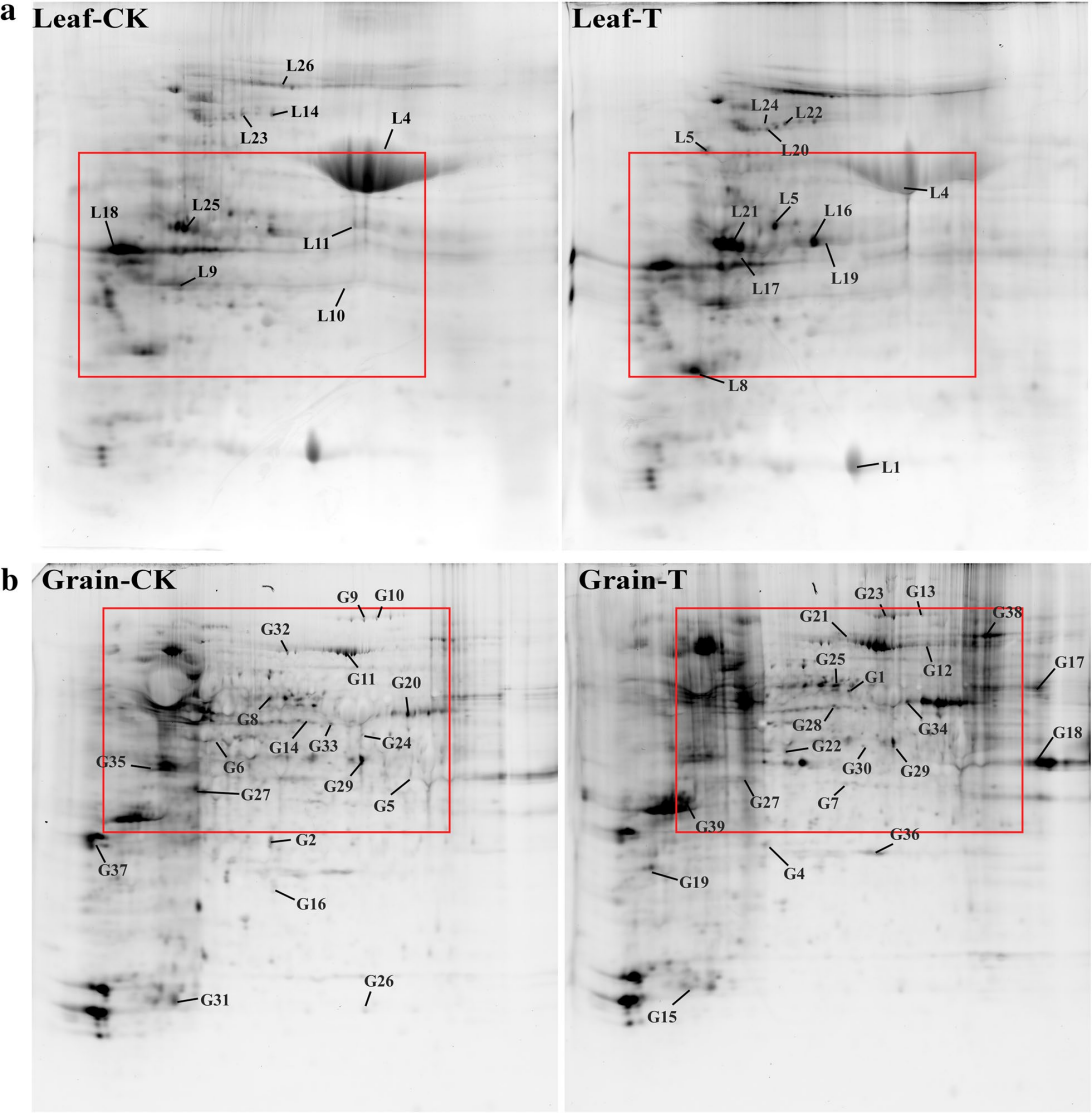
Detection of phosphorylated proteins in 2-DE gels by Pro-Q diamond staining. **a** The image of Pro-Q diamond staining in flag leaves. **b** The image of Pro-Q diamond staining in grains

Through comparison of 2-DE (Fig. 3), Pro-Q Diamond staining (Fig. 5), and tandem mass spectrometry results, 20 and 38 phosphorylated DAP spots in response to water deficit were respectively identified in flag leaves and developing grains, which were individually represented 18 and 38 unique phosphoproteins in flag leaves and developing grains (Table 1). According to the functional categories (Fig. 4a), the phosphorylated proteins in flag leaves were mainly involved in energy metabolism (35%) and photosynthesis (35%), while those in the developing grains mainly participated in stress defense (26.31%), protein metabolism (23.68%), and energy metabolism (18.42%). Most of the phosphorylated proteins in flag leaves were present in chloroplast (80%) (Fig. 4b), and 52.63% grain phosphorylated proteins were located in cytoplasm (Fig. 4c). Thus, drought stress mainly affected photosynthesis and energy metabolism in the flag leaves, and stress/defense, energy metabolism, protein trafficking and degradation in the developing grains.

**Table 1:** Representative phosphorylated proteins identified by MALDI-TOF/TOF-MS and Pro-Q staining

Spot no.	Protein name	Protein score	Peptide count	*p*-value	Subcellular localization	Protein level	Phosphorylation level
*Photosynthesis*							
L1	Ribulose-1,5-bisphosphate carboxylase/oxygenase small subunit	300	18	0.021	Chloroplast	Down	Down
L4	Ribulose-1,5-bisphosphate carboxylase/oxygenase large subunit	1120	30	0.03	Chloroplast	Down	Down
L5	Ribulose-1,5-bisphosphate carboxylase/oxygenase large subunit	695	29	0.024	Chloroplast	Up	Up
L8	Chlorophyll a–b binding protein 8, chloroplastic	111	5	0.041	Chloroplast	Up	Up
L9	Chlorophyll a–b binding protein, chloroplastic	399	10	0.028	Chloroplast	Up	Up
L10	Ribulose bisphosphate carboxylase/oxygenase activase A, partial	363	17	0.029	Chloroplast	Down	Down−
L11	Ribulose bisphosphate carboxylase/oxygenase activase A, partial	402	17	0.042	Chloroplast	Down	Down−
L14	RuBisCO large subunit-binding protein subunit beta	1030	20	0.013	Chloroplast	Down	Down
G1	Ribulose-1,5-bisphosphate carboxylase/oxygenase large subunit, partial	166	10	0.038	Chloroplast	Up	Down
*Energy metabolism*							
L13	Ribulose bisphosphate carboxylase activase B	539	18	0.031	Chloroplast	Up	Up
L16	Fructose-bisphosphate aldolase, cytoplasmic isozyme 1	606	24	0.019	Cytoplasm	Up	Up
L17	Chloroplast fructose-1,6-biphosphate aldolase	490	17	0.035	Chloroplast	Up	Up
L19	ATP synthase subunit	255	20	0.043	Chloroplast	Down	Down
L21	Phosphoglycerate kinase, chloroplastic	916	17	0.028	Chloroplast	Up	Up
L25	V-type proton ATPase subunit B 1	538	36	0.019	Chloroplast	Up	Up
L26	ATP-dependent Clp protease ATP-binding subunit clpA-like protein CD4B	383	38	0.02	Chloroplast	Down	Up
G2	Fructose-bisphosphate aldolase cytoplasmic isozyme	118	12	0.019	Cytoplasm	Up	Up
G4	Triosephosphate-isomerase	619	3	0.018	Cytoplasm	Up	Up
G5	Glyceraldehyde-3-phosphate dehydrogenase	75	14	0.032	Cytoplasm	Down	Up
G6	Enolase	366	18	0.018	Cytoplasm	Down	Down
G7	Pyruvate, phosphate dikinase 1	491	39	0.018	Chloroplast	Up	Up
G8	Dihydrolipoyl dehydrogenase	253	23	0.015	Mitochondrion	Up	Up
G9	Aconitate hydratase	163	25	0.043	Cytoplasm	Up	Up
*Storage substance biosynthesis*							
G10	Sucrose synthase type 2	809	31	0.002	Cytoplasm	Up	Up
G11	Sucrose synthase 2	432	35	0.028	Cytoplasm	Up	Up
G12	Phosphoglucomutase	476	18	0.019	Cytoplasm	Up	Up
*Stress defense*							
G13	Heat shock protein 101	916	40	0.039	Nucleus	Up	Up
G14	17.9 kDa class I heat shock protein-like	224	21	0.017	Cytoplasm	Up	Up
G15	Oxalate oxidase 2	97	5	0.021	Cell-Membrane	Up	Up
G16	Dehydroascorbate reductase	812	15	0.035	Cytoplasm	Up	Down
G17	Disease resistance protein RPP8	48	14	0.022	Cytoplasmic	Up	Up
G18	Peroxidase 1	340	13	0.032	Vacuole	Down	Up
G19	Translationally-controlled tumor protein	552	16	0.022	Cytoplasm	Down	Up+
G31	Hypothetical protein TRIUR3_21260	632	8	0.006	Cytoplasm	Down	Down
G35	Cold regulated protein	210	6	0.021	Cytoplasm	Down	Down
G39	Translationally controlled tumor protein	264	7	0.021	Cytoplasm	Up	Up
*Carbon metabolism*							
L20	Arabinoxylan arabinofuranohydrolase isoenzyme AXAH-II	251	12	0.029	Cell-Membrane	Up	Up
L24	Predicted protein	316	14	0.039	Chloroplast	Down	Up
G24	Basic endochitinase C	130	3	0.029	Extracellular	Up	Down
G28	Formate dehydrogenase	196	3	0.026	Mitochondrion	Up	Up
G30	Glucose and ribitol Dehydrogenase-like protein	264	11	0.032	Cytoplasm	Up	Down
*Protein biosynthesis and degradation*							
L18	Peptidyl-prolylcis-trans isomerase CYP38	371	21	0.015	Chloroplast	Down	Down
L22	Methionine synthase 1 enzyme	391	27	0.032	Cytoplasm	Down	Up
L23	Methionine synthase 1 enzyme	391	27	0.006	Cytoplasm	Down	Up
G20	Globulin-1 S allele	416	16	0.024	Vacuole	Up	Up
G22	Serpin-Z2B	712	18	0.034	Chloroplast	Down	Down
G23	ATP-dependent zinc metalloprotease FTSH 2	53	12	0.027	Chloroplast	Up	Up
G25	Alanine aminotransferase 2	304	22	0.017	Cytoplasm	Down	Up
G27	Serpin 1	500	17	0.041	Extracellular	Up	Down
G32	Putative methionine synthase	157	1	0.014	Cytoplasm	Up	Up
G33	Aspartate aminotransferase	194	9	0.021	Cytoplasm	Down	Up
G34	Globulin 3	489	20	0.017	Vacuole	Down	Up
G37	Elongation factor 1-beta	159	4	0.042	Vacuole	Down	Down

Up: upregulated in phosphorylation level; Down: downregulated in phosphorylation level. Up+: the phosphoproteins were only identified in drought treatment. Down−: the phosphoproteins were only identified in CK

Associated with 2-DE gels and Pro-Q Diamond gel staining, some isoforms of DAPs resulted from phosphoprotein were found. In the flag leaves, ribulose-1,5-bisphosphate carboxylase/oxygenase large subunit had three different isoforms (L4, L5 and L6). Meanwhile, the phosphorylation of both L4 and L5 was detected by Pro-Q Diamond. In the developing grains, ribulose-1,5-bisphosphate carboxylase/oxygenase large subunit (G1 and G59) and fructose-bisphosphate aldolase cytoplasmic isozyme (G2 and G3) also had different isoforms, of which G2 and G3 was individually phosphorylated (Table 1).

### Hierarchical clustering analysis of DAPs and phosphorylated proteins in response to water deficit

**Fig. 6:**
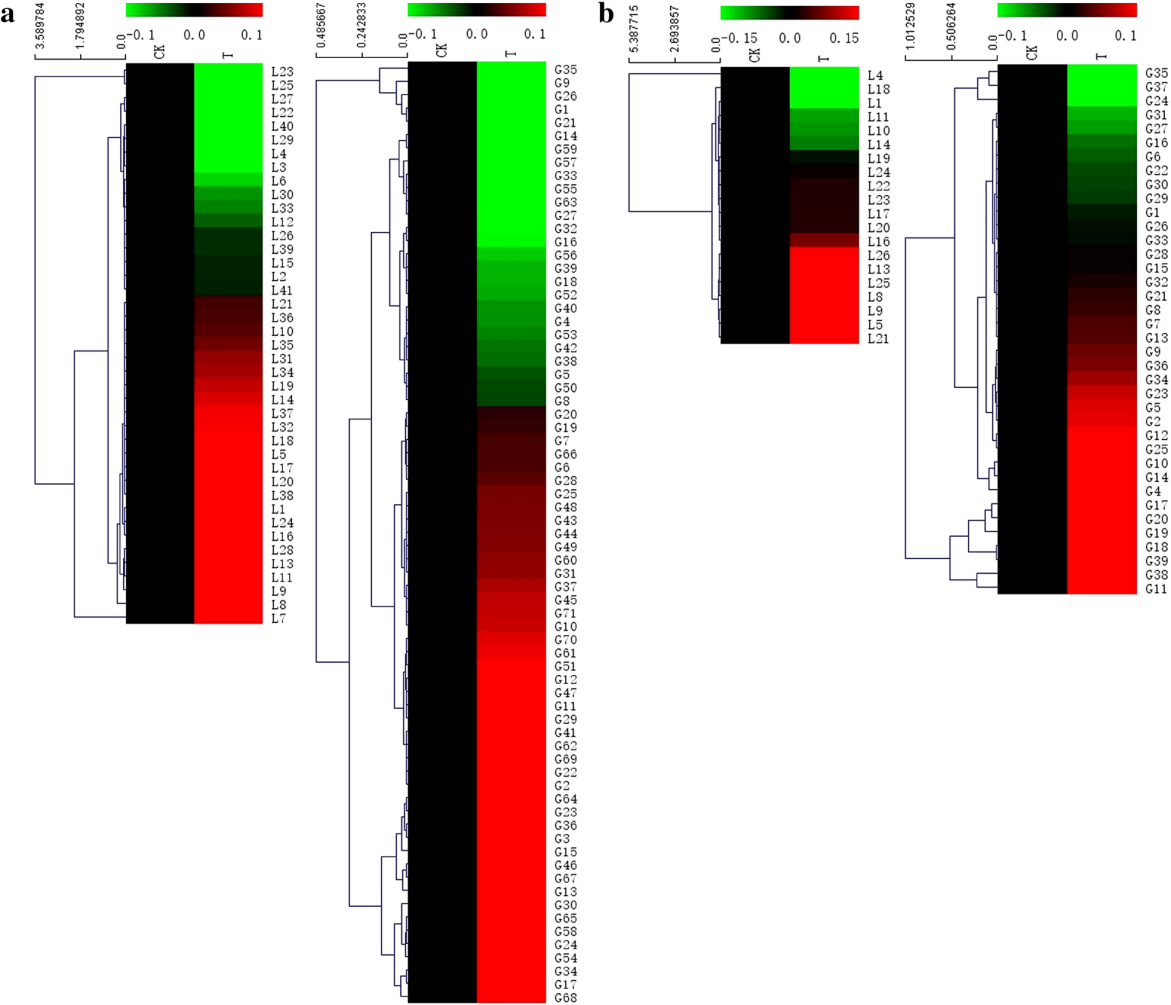
Hierarchical clustering analysis of the DAP spots and phosphorylated proteins identified from flag leaves and developing grains. **a** Hierarchical clustering of all DAP spots from flag leaves and developing grains in protein level; **b** hierarchical clustering of phosphorylated proteins from flag leaves and developing grains in phosphorylation level. The upregulation and downregulation are indicated are indicated in red and green, respectively

Hierarchical clustering analysis was conducted to reveal the accumulation patterns of DAPs and phosphorylated proteins under water deficit (Fig. 6). The results showed that 24 (58.54%) and 45 (63.38%) DAPs from flag leaves and developing grains were significantly upregulated, respectively (Fig. 6a). In the flag leaves, the upregulated DAPs were mainly related to energy metabolism while the downregulated DAPs were majorly involved in photosynthesis. In the developing grains, stress defense and energy metabolism related DAPs were drastically increased whereas those related to protein metabolism were downregulated.

The accumulation patterns of the phosphorylated proteins under water deficit showed that 13 (65%) and 25 (65.78%) phosphoproteins were upregulated in the flag leaves and grains, respectively (Fig. 6b). We found that most phosphoproteins involved in carbon metabolism and energy metabolism were upregulated in flag leaves, but some important phosphoproteins participating in photosynthesis were downregulated. In the developing grains, the modification levels of those related to energy metabolism, stress defense and storage substance biosynthesis increased. Thus, most phosphoproteins involved in photosynthesis were both downregulated whereas those related to energy metabolism, drought stress response and storage substance biosynthesis were generally upregulated in both phosphorylated modification level and protein level. Particularly, ribulose bisphosphate carboxylase/oxygenase activase A (L10 and L11) was only identified in flag leaves under well-watered group and translationally-controlled tumor protein (G19) was only identified in the developing grains under drought treatment group. This suggests that water deficit caused the dephosphorylation of ribulose bisphosphate carboxylase/oxygenase activase A and specific phosphorylation of translationally-controlled tumor protein.

### Verification of phosphorylated proteins by phosphorylated site prediction and Western blotting

**Fig. 7:**
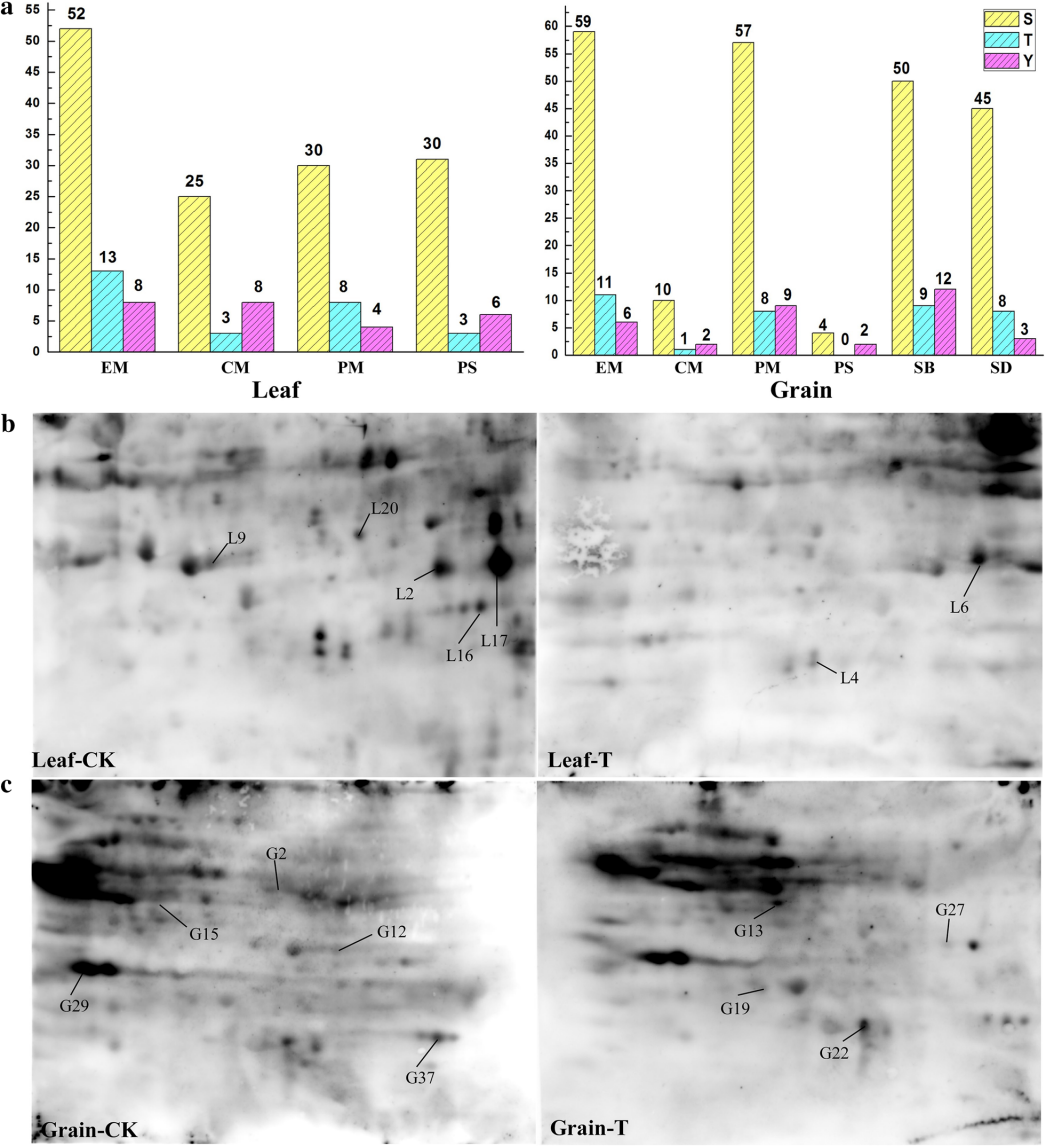
Prediction of phosphorylated site by phosphorylation site software and verification of phosphoproteins by Western-blotting. **a** The numbers of serine/threonine/tyrosine predictive phosphorylation sites in main functional groups in flag leaf and grain. S: phosphosites in serine residues, T: phosphosites threonine residues, Y: phosphosites tyrosine residues. EM: energy metabolism; CM: carbon metabolism; PM: protein metabolism; PS: photosynthesis; SB: Starch biosynthesis; SD: stress defense. **b** Western-blotting analysis in leaves of CK and drought treatment group, respectively. **c** Western-blotting analysis in the grains of CK and drought treatment group. All the gels used to perform Western blotting were chosen from red box in Fig. 5

To provide further supporting for the presence of the phosphorylated proteins identified by Pro-Q Diamond staining, the phosphorylated sites of all 58 phosphorylated proteins were predicted by NetPhos 3.1 Server. High scores (≥ 0.90) were set to increase the accuracy of prediction and the results are shown in Additional file 1: Table S4A, B. In total, 58 phosphorylated proteins contained 543 phosphorylated peptides, of which 191 were from flag leaves and 352 from developing grains (Additional file 2: Fig. S1a). Combined with functional categories, we found that phosphorylated proteins related to energy metabolism contained the most phosphorylated sites, including 73 (38.22%) from flag leaves and 76 (21.59%) from grains (Fig. 7a). The second largest category was those related to protein metabolism, which accounted for 26.70% (51) in flag leaves and 21.02% (74) in developing grains. Generally, both flag leaves and grains had similar percentages of serine (74%), threonine (14%) and tyrosine (12%) phosphorylation (Additional file 2: Fig. S1b, d). Thus, consistent with the previous reports (Zhang et al. 2014a, b), serine phosphorylation had the highest percentage among three phosphorylated amino acid residues.

Western blotting was performed to further verify the identified phosphorylated proteins using an Anti-Phosphoserine/threonine/tyrosine monoclonal antibody (Fig. 7b, c). Through comparison of Western blotting results with 2-DE and Pro-Q Diamond gels, the results showed that 16 phosphorylated proteins, including 7 from flag leaves (Fig. 7b) and 9 from developing grains (Fig. 7c) were confirmed to be phosphorylated, such as ribulose-1,5-bisphosphate carboxylase/oxygenase large subunit, phosphoglycerate kinase, chloroplastic and formate dehydrogenase etc. These phosphorylated proteins shown in Fig. 7b, c were well corresponding to Pro-Q Diamond staining results listed in Table 1.

## Discussion

### Phosphorylated proteins participating in photosynthesis regulation

Photosynthesis is an essential metabolic process that directly impacts carbohydrate synthesis, grain development and yield formation. Phosphorylation of chloroplast membrane proteins is ultimately responsible in plants, responding to changes in incident light and redox poise (Allen 1983, 1992a). Light-harvesting complex II (LHCII), the chloroplast light-harvesting chlorophyll a/b-binding complex, is a major substrate protein of phosphorylation, which binds perhaps half of the chlorophyll in nature. It is known that the light-harvesting complex changes its allegiance upon phosphorylation. Phosphorylated proteins have a great effect on enzyme structure, specificity or activities such as glycogen phosphorylase and isocitrate dehydrogenase and therefore play a key role in photosynthesis (Allen 1992b). In this study, we identified several phosphorylated proteins associated with photosynthesis in flag leaves, including a series of ribulose-1,5-bisphosphate carboxylase/oxygenase (Rubisco) proteins, chlorophyll a–b binding proteins (CBPs, L8–L9) and ribulose bisphosphate carboxylase/oxygenase activase (L10–L13). One phosphorylated Rubisco protein in response to water deficit was also identified in the developing grains (G1) (Table 1). This indicates that photosynthesis also happened in grains when subjected to drought stress, which could be beneficial for grain development. Meanwhile, we found that most phosphoproteins involved in photosynthesis were downregulated in protein modification level as well as protein level, which could be responsible for the reduction of photosynthesis and grain yield.

Rubisco initiates both the photosynthetic carbon reduction and photorespiratory carbon oxidation cycles (Bowes 1991). Rubisco large subunit binding protein (RSBL) is involved in the assembly of Rubisco in higher plant chloroplasts, which can be dissociated to monomeric subunits with binding ATP, but re-attached by removal of ATP (Musgrove and Ellis 1986). We found that both the expression level and the phosphorylated level of this protein are downregulated under drought treatment (Table 1), suggesting that the assembly of Rubisco and photosynthesis were heavily restrained under drought stress. Rate of photosynthesis is heavily dependent on the activity of Rubisco proteins (Chaitanya et al. 2002). Drought normally diminishes the biochemical capacity for carbon assimilation and utilization (Reddy et al. 2004). We found that the phosphorylated level of Rubisco was downregulated in flag leaves, suggesting that drought inhibits photosynthesis mainly through reducing phosphorylation modification level of Rubisco. Rubisco activase has the function of activating Rubisco (Feller et al. 1998). It was only identified by Pro-Q diamond staining in well-watered flag leaves, suggesting that this protein was dephosphorylated duo to drought stress. Rubisco activase mediates Rubisco, which is ATP-dependent (Feller et al. 1998). We found that drought heavily inhibited phosphorylation of Rubisco activase and affected the activity of Rubisco activase. Thus, the function of photosynthesis was severely disturbed, ultimately leading to grain yield decrease.

CBP, a light-harvesting complex serves as a light receptor and has diverse functions (Bassi et al. 1992). It captures and delivers excitation energy to photosystems. In this study, both phosphorylation and protein levels of CBP and CBP 8 were upregulated under drought stress in the leaf, consistent with the recent report (Bian et al. 2017). This suggests that a few phosphorylated proteins such as CBP were upregulated to alleviate the influences on photosynthesis when suffered from drought stress.

### Phosphorylated proteins involved in energy metabolism

Energy metabolism is basic radical cellular activity to maintain normal growth and development, which involves in three respiratory pathways: glycolysis, themitochondrial electron transport chain, and TCA cycle. These pathways are essential for energy supply to numerous cellular functions and greatly depend on phosphorylation of proteins (Fernie et al. 2004). In this study, energy metabolism related phosphorylated proteins accounted for a great proportion in function categories in both flag leaves and grains. Among them, 5 DAPs involved in glycolysis with an upregulation at 20 DPA of grain development in both protein and phosphorylaed modification levels, including fructose-bisphosphate aldolase (FBA) cytoplasmic isozyme (G2-G3), triosephosphate isomerase (G4), glyceraldehyde-3-phosphate dehydrogenase (GAPDH, G5), enolase (G6) and pyruvate phosphate dikinase (G7) (Table 1). In the flag leaf, two FBAs (L16–L17) were upregulated, one of which located in cytoplasm and the other in chloroplast. This indicated that drought expedited energy metabolism in both flag leaf and grain development, consistent with the previous reports (Caruso et al. 2009; Budak et al. 2013).

Two enzymes (dihydrolipoyl dehydrogenase and aconitate hydratase, G8 and G9) involved in TCA cycle were identified in this study. Both of them were up-regulated at protein and phosphoprotein level at 20 DPA of developing grains. The pyruvate dehydrogenase complex (PDC), consisting of dihydrolipoyl dehydrogenase, catalyzes the irreversible conversion of pyruvate, coenzyme A and NAD+ into CO_2_, NADH and acetyl-CoA (Patel and Roche 1990). Obviously, TCA cycle has similar trend to glycolysis, and plants increased TCA cycle and glycolysis metabolism in the developing grains to provide sufficient energy for starch biosynthesis. Similar report also showed that the ATP/ADP ratio was significantly increased in spring wheat plants under drought stress (Chen et al. 2004).

### Starch biosynthesis regulation via protein phosphorylation

Starch biosynthesis needs abundant triosephosphates provided by photosynthesis in the early grain development stages (Tschiersch et al. 2011). A large number of phosphorylated enzymes related to starch biosynthesis were needed to transform intermediates of photosynthesis into starch (Tschiersch et al. 2011). In this study, we identified two key enzymes involved in starch biosynthesis: sucrose synthase (G10, G11) and phosphoglucomutase (PGM, G12). Phosphorylation of these proteins could be induced by drought stress (Zhang et al. 2014a) and high-nitrogen (Zhen et al. 2017). At the beginning of starch biosynthesis, ADP glucose pyrophosphorylase (ADPase) catalyzes the first committed step of the starch biosynthetic pathway, converting glucose 1-phosphate and ATP to glucosyl moiety of ADP (ADPG) and pyrophosphate (Tschiersch et al. 2011). Then, ADPG is metabolized by sucrose synthase (SS) as it arrives in the cytosol of endosperm cells (Tomlinson and Denyer 2003). SS catalyzes starch biosynthesis by transferring the ADPG glucose to the non-reducing end of an existing α-1,4-glucan chain. It has reported that loss of SSII in *T. aestivum* and *H. vulgare* endosperms causes a drastic reduction in amylopectin synthesis and abolishes binding of SSI, SBEIIa, and SBEIIb to the starch granules (Yamamori et al. 2000). We found that the phosphorylation level as well as protein level of SS was upregulation under drought stress, which could enhance enzyme activity and protein–protein interactions (Tetlow et al. 2004), and expedite starch biosynthesis and grain development under water deficit environment.

PGM has function of interconverting glucose-1-phosphate and glucose-6-phosphate, with glucose 1,6-bisphosphate as a cofactor (Ray et al. 1983). It has function that the transfer of a phosphoryl group from the itself to glucose 1-phosphate, forming glucose 1,6-bisphosphate (Najjar and Pullman 1954). The glucose-1-phosphate can be supplied in chloroplast phosphoglucomutase through the reductive pentose phosphate pathway (Smith and Martin 1993). However, it could be directly supplied by cytosol (Tyson and Rees 1988) or synthesized with the catalysis of a plastidial phosphoglucomutase in nonphotosynthetic tissues (Hill and Smith 1991). The biochemistry reaction catalyzed by PGM is a principal distinction in carbon and starch synthesis pathways. According to our results, PGM was unregulated in both phosphorylation and protein levels at 20 DPA of developing grains under drought stress conditions, which might boost to transform glucose-6-phosphate to glucose-1-phosphate. Thus, grain filling was advanced by drought stress due to acceleration of starch biosynthesis, consistent with energy metabolism in the developing grains.

### Protein phosphorylation regulating plant drought stress response

During grain development, many hydrophobic proteins were gradually synthesized and accumulated to protect cells from various adverse environments (Zhang et al. 2014a). Drought has a severe limitation on wheat growth and yield formation. In this study, several proteins under drought conditions were found to be phosphorylated and up-regulated as well as in protein level, including 2 heat shock proteins (G13 and G14), one manganese ion binding protein (oxalate oxidase 2, G15), dehydroascorbate reductase (DHAR, G16), disease resistance protein RPP8 (G17), peroxidase 1 (G18) and translationally-controlled tumor protein (TCTP, G19). Particularly, the phosphorylation of TCTP was specifically induced by drought stress, suggesting its important roles in resistance to drought stress. TCTP is a calcium-binding protein (Sanchez et al. 1997) as well as tubulin-binding protein that associates with microtubules (Vandre et al. 1991). The expression level of plant TCTP could increase in response to abiotic stresses such as salt (Santa Brígida et al. 2014) and drought (Kim et al. 2012). TCTP could be phosphorylated at serine residues 46 and 64 by a polo-like protein kinase that regulates spindle function (Jung et al. 2004).

Plants are easily damaged by accumulated ROS under drought stress, including singlet oxygen, superoxide radical (O^2−^), hydrogen peroxide (H_2_O_2_), and hydroxyl radical (OH) (Smith and Martin 1993). Many biological processes such as stress responses, hormone signaling, cell growth, and development, are related to ROS (Pei et al. 2000). Therefore, it is highly important for plants to scavenge high levels of ROS when suffered from drought. It is known that the ascorbate–glutathione (AsA–GSH) cycle is particularly important in plant antioxidant defense mechanism. Four anti-oxidative enzymes participate in ascorbate–glutathione cycle, including ascorbate peroxidase (APX), monodehydroascorbate reductase, dehydroascorbate reductase (DHAR), and glutathione reductase. In this study, a DHAR was identified in the drought treatment group at 20 DPA of developing grains, which is an indispensable enzyme functioning in the regeneration of ascorbate and glutathione that scavenges hydrogen peroxide and reduces it to water, with concomitant oxidation of NADPH (Eltayeb et al. 2006). Meanwhile, its phosphorylation and protein levels were up-regulated under drought stress, in accordance with our conjecture that drought stress may transform into oxidative stress, and proteins phosphorylated modification plays a key role to respond drought stress. The other anti-oxidative enzymes peroxidase 1 was also upregulated in grains, which is a key capable enzyme of removing ROS (De Pinto et al. 2006). Their phosphorylation could enhance ROS scavenging and drought tolerance.

Under abiotic stresses, maintaining proteins in their functional conformations and preventing protein misfolding are highly important for cell survival (Kim et al. 2013). Heat-shock proteins (Hsps) function in protein folding, assembly, translocation and degradation as well as assistance in protein refolding under stress conditions (Boston et al. 1996). Thus, Hsp family plays an imperative role in protecting plants against stress by re-establishing normal protein structure and cellular homeostasis (Bukau and Horwich 1998). Up-regulation of two Hsps was identified with enhancement in modification and expression level in this study. Hsp 101 can augment *Arabidopsis* resistance to thermo stress (Queitsch 2000). To our knowledge, the mechanism of HSP 101 functioned in adverse response is still not clear. Its phosphorylation was also found in wheat developing grains (Guo et al. 2012). The 17.9 kDa class I heat shock protein-like was found to be upregulated under drought stress in both phosphorylation and protein accumulation levels. We speculate that the phosphorylation of Hsp family may be an important mechanism in response to drought stress.

## Conclusion

This study identified 58 phosphorylated proteins among 112 differentially accumulated proteins in response to water deficit at 20 days of postanthesis, including 20 in the flag leaves and 38 in the developing grains. The phosphorylated proteins from flag leaves mainly involved in photosynthesis, carbohydrate metabolism and energy metabolism while those from developing grains mainly participated in detoxification and defense, protein metabolism, carbohydrate metabolism and energy metabolism. Most phosphoproteins related to photosynthesis in flag leaves were significantly downregulated such as ribulose-1,5-bisphosphate carboxylase/oxygenase large subunit, ribulose bisphosphate carboxylase/oxygenase activase, leading to significant decrease in starch biosynthesis and grain yield. On the contrary, most phosphoproteins associated with drought stress response as well as energy metabolism were upregulated in the developing grains, demonstrating the important roles of protein phosphorylation in resisting to drought, and expediting starch biosynthesis and grain filling. Our results provide new evidence for protein phosphorylated modification how to regulate plant drought tolerance and grain development.

## Additional files

**Additional file 1: Table S1.** Agronomic trait changes of Zhongmai 175 under drought stress. **Table S2A.** DAPs from flag leaf samples identified by MALDI-TOF/TOF-MS. **Table S2B.** Identified DAP spots from 2-DE maps of flag leaves in Zhongmai 175 under drought stress treatment in combination with MALDI-TOF/TOF-MS data. **Table S3A.** DAPs from developing grain samples identified by MALDI-TOF/TOF-MS. **Table S3B.** Identified DAP spots from Zhongmai 175 developing grain under drought stress treatment from 2-DE maps in combination with MALDI-TOF/TOF-MS data. **Table S4A.** Phorphorylated site prediction of the identified phosphoproteins in flag leaf by NetPhos 3.1 Server. **Table S4B.** Phorphorylated site prediction of the identified phosphoproteins in developing grains by NetPhos 3.1 Server.

**Additional file 2: Fig. S1.** Statistics of predicted phosphorylation sites. (a) The numbers of serine/threonine/tyrosine phosphorylated sites in flag leaves and developing grains, respectively. (b), (c) and (d) represented the percentage of serine residues/threonine residues/tyrosine residues phosphorylation in flag leaves, developing grains and all phosphorylated sites, respectively.

## Abbreviations

2-DE: two-dimensional electrophoresis
AGPase: ADP glucose pyrophosphorylase
APX: ascorbate peroxidase
CBPs: chlorophyll a–b binding proteins
DAPs: differentially accumulated proteins
DHAR: dehydroascorbate reductase
DHAR: dehydroascorbate reductase
DPA: days postanthesis
FBA: fructose-bisphosphate aldolase
GAPDH: glyceraldehyde-3-phosphate dehydrogenase
Hsps: heat-shock proteins
MALDI-TOF/TOF-MS: matrix-assisted laser desorption/ionisation time-of-flight/time-of-flight mass spectrometry
NCBI: National Center for Biotechnology Information
PDC: pyruvate dehydrogenase complex
PGM: phosphoglucomutase
PTMs: protein posttranslational modifications
ROS: reactive oxygen species
Rubisco: ribulose-1,5-bisphosphate carboxylase/oxygenase
SDS–PAGE: sodium dodecyl sulfate–polyacrylamide gel electrophoresis
SS: starch synthases
TCTP: translationally-controlled tumor protein
