# Supplementary figures and images for: Identification of phosphorylation proteins in response to water deficit during wheat flag leaf and grain development
Source: Bot Stud. 2018 Dec 8;59:28. doi: 10.1186/s40529-018-0245-7 (PMC6286713; doi:10.1186/s40529-018-0245-7)

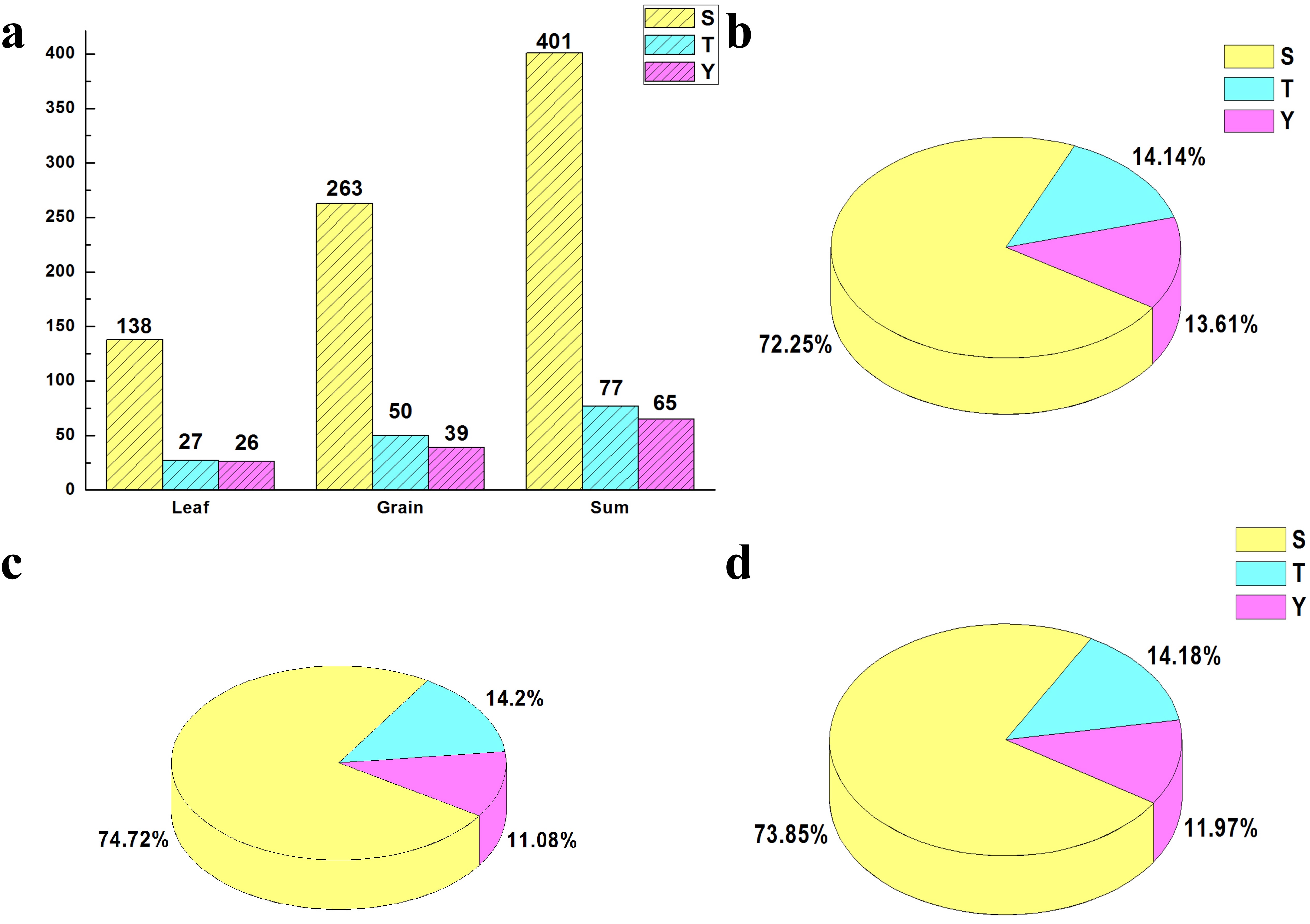

Supplement: Supplementary file 2 — Additional file 2: Fig. S1. Statistics of predicted phosphorylation sites. (a) The numbers of serine/threonine/tyrosine phosphorylated sites in flag leaves and developing grains, respectively. (b), (c) and (d) represented the percentage of serine residues/threonine residues/tyrosine residues phosphorylation in flag leaves, developing grains and all phosphorylated sites, respectively. [file 40529_2018_245_MOESM2_ESM.jpg]
